# Supplementary material for: HCV Genetic Diversity Can Be Used to Infer Infection Recency and Time since Infection
Source: Viruses. 2020 Oct 31;12(11):1241. doi: 10.3390/v12111241 (PMC7692400; doi:10.3390/v12111241)
Supplement: Supplementary file 1 [file viruses-12-01241-s001.pdf]

Supplementary materials

Supplementary material S1: Full protocol for preparation and sequencing of samples for study 1

RNA extraction

RNA was extracted from 500µl of peripheral blood plasma from HCV-infected patients following standard procedures according to the manufacturer's instructions (EasyMag; BioMerieux), and eluted in 50µl.

cDNA synthesis and amplification

The methods used for this step were continuously updated, resulting in a total of five different method variants, summarised in Supplementary table 1.

Version 1: Extracted RNA (4µl) was reverse transcribed in a total of 20µl with 250µM of each forward and reverse primer using the PrimeScript One Step RT-PCR Kit (TaKaRa Bio Inc) and followed by the first round PCR (9.1kb), all according to the manufacturer's instructions. Nested-PCRs with 250µM of each primer for the full length region were run with 1µl of the first amplicon in a total of 40µl using the Phusion Hot Start II High Fidelity DNA Polymerase (Thermo Scientific) according to the manufacturer's instructions.

Version 2+3: Extracted RNA (4µl) was reverse transcribed in a total of 10µl using the SuperScript IV Reverse Transcriptase according to the manufacturer's instructions, but without DTT (Thermo Fisher Scientific). First-Round PCR were performed with 4µl of the cDNA product in a total of 20µl with 250µM of each forward and reverse primer using the Phusion Hot Start II High-Fiedlity DNA Polymerase (Thermo Scientific) according to the manufacturer's instructions. Nested-PCRs with 250µM of each Primer for the two half-length regions (5'end and 3'end) were run with 1µl of the first amplicon in a total of 40µl using the Phusion Hot Start II High Fidelity DNA Polymerase (Thermo Scientific) according to the manufacturer's instructions. The two half-length regions overlap by at least 300bp.

Version 4+5: Extracted RNA (4µl) was reverse transcribed in a total of 10µl using the SuperScript IV Reverse Transcriptase according to the manufacturer's instructions, but without DTT (Thermo Fisher Scientific). One-Round PCR were performed with 4µl of the cDNA product in a total of 20µl with 250µM of each forward and reverse primer for the full length region or for the two half-length regions (5'end and 3'end) using the Phanta Max Super-Fidelity DNA Polymerase (Vayzme) according to the manufacturer's instructions.

NGS

Pooled nested-PCR products were quantified with the QuantiFluor ONE dsDNA system (Promega, Madison, WI) on the Quantus Fluorometer (Promega) and diluted to 0.2 ng/µl. Sequencing libraries were constructed with the NexteraXT protocol (Illumina, San Diego, CA). Individual samples were dual indexed during library preparation and pooled for sequencing. Libraries were sequenced on a MiSeq (Illumina) for 1 x 150 cycles with version 3 reagents and the 'FASTQ only' workflow. Samples were demultiplexed using MiSeq Reporter v2.6.2.

Supplementary Table S1: Summary of preparations methods for sequencing for study 1.

<table>
<tr><td></td><td>Version</td><td>1</td><td>2</td><td>3</td><td>4</td><td>5</td></tr>
<tr><td></td><td>Number of samples</td><td>1</td><td>13</td><td>3</td><td>24</td><td>12</td></tr>
<tr><td>cDNA</td><td>Kit/enzyme</td><td rowspan="2">PrimeScript One Step RT-PCR Kit</td><td colspan="4">SuperScript IV Reverse Transcriptase</td></tr>
<tr><td rowspan="3">1st PCR</td><td>Kit/enzyme</td><td colspan="2">Phusion Hot Start II High-Fidelity DNA Polymerase</td><td colspan="2">Phanta Max Super-Fidelity DNA Polymerase</td></tr>
<tr><td>Spanning nucleotides[a]</td><td colspan="2">130-9303</td><td>130-9602</td><td colspan="2">150-9602</td></tr>
<tr><td>Amplicon (length)</td><td colspan="3">One (9.1kb)</td><td>Two (5.3kb + 4.4kb)</td><td>One (9.4kb)</td></tr>
<tr><td rowspan="3">Nested PCR</td><td>Kit/enzyme</td><td colspan="3">Phusion Hot Start II High Fidelity DNA Polymerase</td><td colspan="2" rowspan="3">N/A</td></tr>
<tr><td>Spanning nucleotides[a]</td><td colspan="3">145-9301</td></tr>
<tr><td>Amplicon (length)</td><td>One (9.1kb)</td><td colspan="2">Two (5.3kb + 4.1kb)</td></tr>
</table>

[a] Reference genome: H77 (GenBank accession number NC 004102)

Supplementary Table S2: Scores for average pairwise diversity calculated over all codon positions, and over various regions of the open reading frame, with associated coefficients from linear regression (AUC = area under the ROC curve, adj. R^2 = adjusted R^2, MAE = mean absolute error).

			Score		Coefficients	
**Region of the open reading frame**		**AUC**	**adj. R^2**	**MAE**	**β [years]**	**α [years]**
Whole open reading frame		0.76	0.33	1.67	277.52	0.58
Gene	*C*	0.82	0.27	1.84	293.70	1.16
	E1	0.71	0.08	2.24	93.15	1.78
	E2	0.85	0.46	1.49	232.79	0.71
	p7	0.75	0.32	1.71	331.71	0.85
	NS2	0.82	0.56	1.39	404.48	0.32
	NS3	0.77	0.16	2.06	153.21	1.31
	NS4A	0.65	0.00	2.19	28.71	2.06
	NS4B	0.66	0.09	2.20	82.57	1.71
	NS5A	0.81	0.34	1.69	238.24	0.80
	NS5B	0.82	0.52	1.52	519.20	0.21
Region (~500 amino acid codons)	1-502	0.81	0.30	1.74	197.76	1.15
	251-752	0.83	0.36	1.60	215.85	0.89
	503-1004	0.85	0.48	1.44	353.70	0.42
	753-1254	0.80	0.36	1.63	325.34	0.49
	1005-1506	0.76	0.17	2.03	168.30	1.23

	1255-1756	0.70	0.10	2.15	99.53	1.57
	1507-2008	0.69	0.12	2.08	110.92	1.52
	1757-2258	0.76	0.35	1.69	260.16	0.82
	2009-2510	0.82	0.38	1.61	257.28	0.69
	2259-2760	0.79	0.25	1.85	224.96	0.97
	2511-3011	0.80	0.49	1.57	542.51	0.19
Region (~1000 amino acid codons)	1-1004	0.84	0.41	1.53	286.77	0.68
	502-1506	0.78	0.30	1.73	265.54	0.69
	1005-2008	0.76	0.15	2.03	144.72	1.33
	1507-2510	0.74	0.23	1.88	182.23	1.05
	2009-3011	0.86	0.47	1.47	387.21	0.27
Combined genes	*E2*, *p7*, *NS2*	0.85	0.54	1.38	320.38	0.39
	E2, *NS2*	0.85	0.53	1.39	302.87	0.44

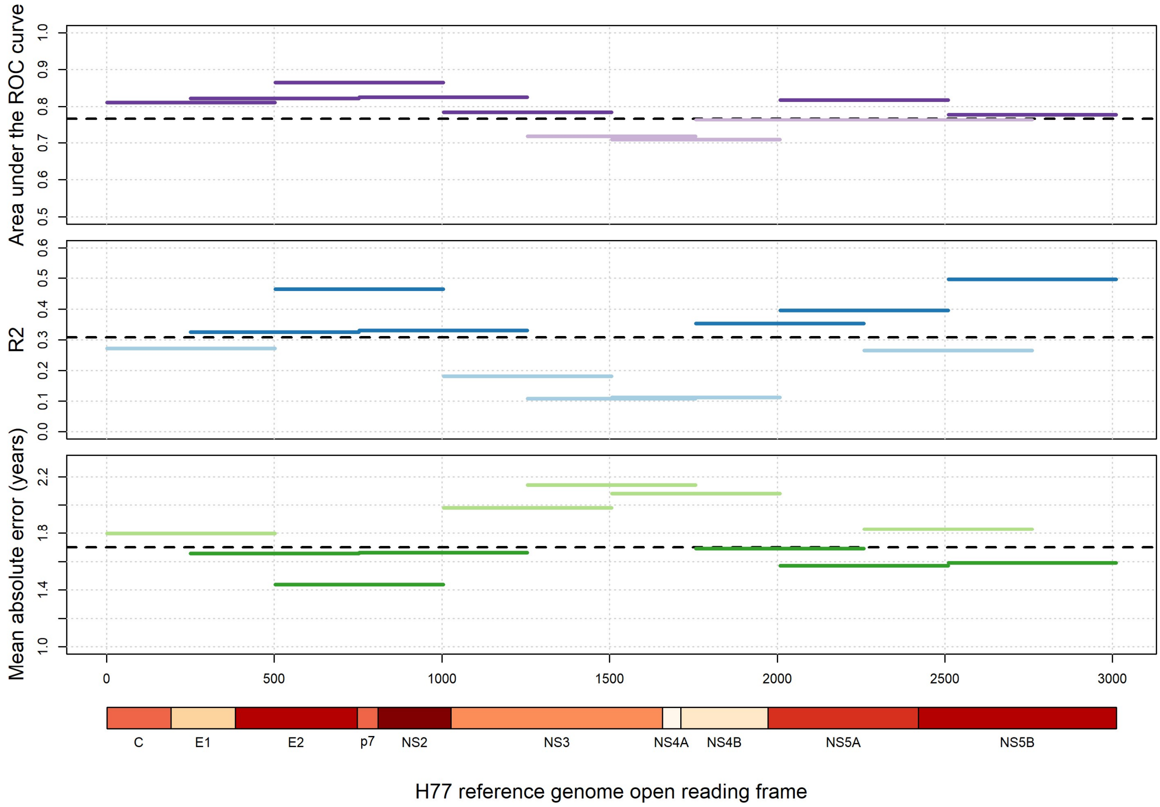

Supplementary Figure S1: Area under the ROC curve, R^2, and mean absolute error across the HCV open reading frame, third codon positions. The HCV open reading frame was split into 11 overlapping regions of approximately 500 amino acid codons, and average pairwise diversity (APD) was calculated over individual regions, using the third codon positions. Regions were tested for their ability to categorise infection as recent (<1 year) or chronic (top), their correlation with time since infection (middle), and their ability to infer time since infection (bottom). Black dashed lines show the

respective values for APD calculated over the whole open reading frame. A similar analysis was performed with diversity calculated over each gene in turn. The HCV genome is shown along the x-axis, with genes colour-coded for a composite (z-score sum) of all three outcome scores. Darker red indicates a better overall performance. Numbers along the x-axis refer to amino acid positions of the H77 reference genome.

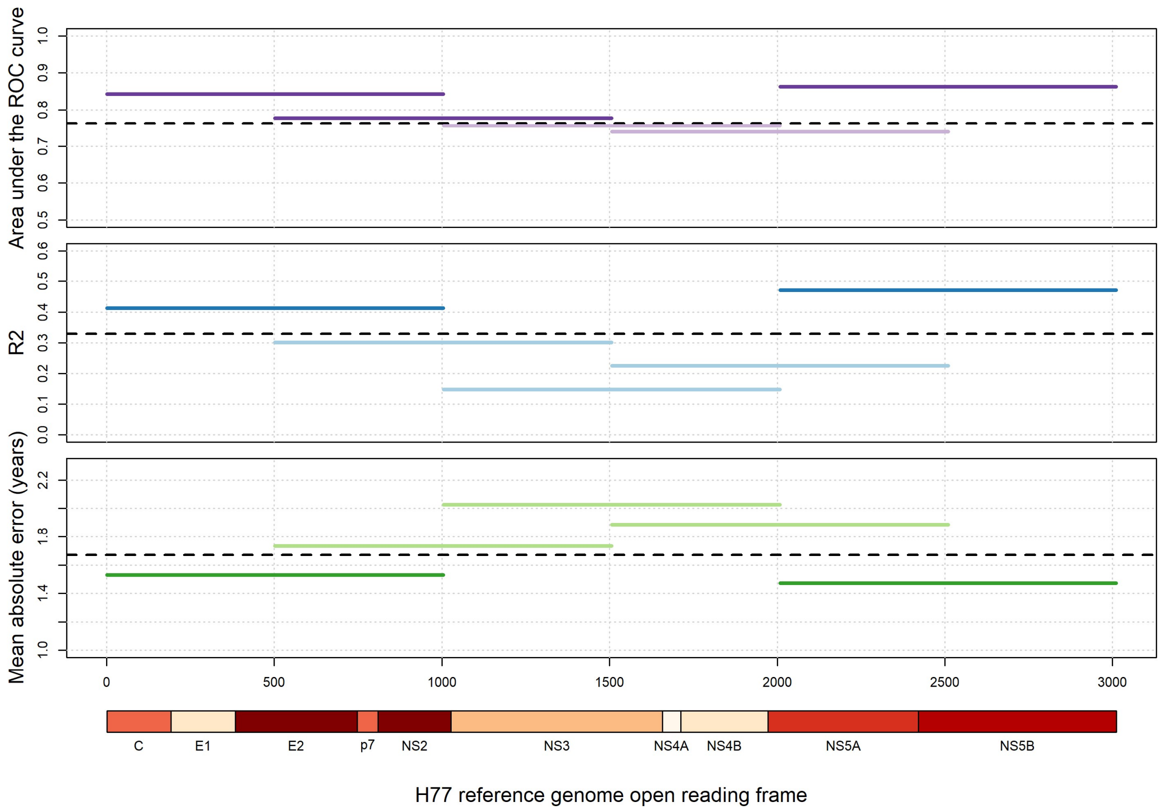

Supplementary Figure S2: Area under the ROC curve, R^2, and mean absolute error across the HCV open reading frame, all codon positions. The HCV open reading frame was split into 5 overlapping regions of approximately 1000 amino acid codons, and average pairwise diversity (APD) was calculated over individual regions, using all codon positions. Regions were tested for their ability to categorise infection as recent (<1 year) or chronic (top), their correlation with time since infection (middle), and their ability to infer time since infection (bottom). Black dashed lines show the respective values for APD calculated over the whole open reading frame. A similar analysis was performed with diversity calculated over each gene in turn. The HCV genome is shown along the x-axis, with genes colour-coded for a composite (z-score sum) of all three outcome scores. Darker red indicates a better overall performance. Numbers along the x-axis refer to amino acid positions of the H77 reference genome.

Supplementary Equation S1: Z-score for converting outcome scores to be summed into a combined single score.

$$\text{z-score} = \frac{x - \bar{x}}{sd(x)}$$

where:

$$x = \text{outcome score for the gene} \quad \text{(s.1)}$$

$$\bar{x} = \text{mean}(x) \text{ over all genes}$$

$$sd = \text{standard deviation over all genes}$$
